# The anxiety and ethanol intake controlling GAL5.1 enhancer is epigenetically modulated by, and controls preference for, high-fat diet

**DOI:** 10.1007/s00018-020-03705-6

**Published:** 2020-12-12

**Authors:** Andrew McEwan, Johanna Celene Erickson, Connor Davidson, Jenny Heijkoop, Yvonne Turnbull, Mirela Delibegovic, Christopher Murgatroyd, Alasdair MacKenzie

**Affiliations:** 1 School of Medicine, Medical Sciences and Nutrition, Institute of Medical Sciences, Foresterhill, University of Aberdeen, Aberdeen, AB25 2ZD Scotland UK; 2 Manchester Metropolitan University, Manchester, UK

**Keywords:** GAL5.1 enhancer sequence, CRISPR genome editing, Galanin, EGR1, Protein Kinase C, Gene regulation, 5mC/5hmC content, Early life stress, Fat preference, Anxiety and substance abuse

## Abstract

Excess maternal fat intake and obesity increase offspring susceptibility to conditions such as chronic anxiety and substance abuse. We hypothesised that environmentally modulated DNA methylation changes (5mC/5hmC) in regulatory regions of the genome that modulate mood and consumptive behaviours could contribute to susceptibility to these conditions. We explored the effects of environmental factors on 5mC/5hmC levels within the GAL5.1 enhancer that controls anxiety-related behaviours and alcohol intake. We first observed that 5mC/5hmC levels within the GAL5.1 enhancer differed significantly in different parts of the brain. Moreover, we noted that early life stress had no significant effect of 5mC/5hmC levels within GAL5.1. In contrast, we identified that allowing access of pregnant mothers to high-fat diet (> 60% calories from fat) had a significant effect on 5mC/5hmC levels within GAL5.1 in hypothalamus and amygdala of resulting male offspring. Cell transfection-based studies using GAL5.1 reporter plasmids showed that 5mC has a significant repressive effect on GAL5.1 activity and its response to known stimuli, such as EGR1 transcription factor expression and PKC agonism. Intriguingly, CRISPR-driven disruption of GAL5.1 from the mouse genome, although having negligible effects on metabolism or general appetite, significantly decreased intake of high-fat diet suggesting that GAL5.1, in addition to being epigenetically modulated by high-fat diet, also actively contributes to the consumption of high-fat diet suggesting its involvement in an environmentally influenced regulatory loop. Furthermore, considering that GAL5.1 also controls alcohol preference and anxiety these studies may provide a first glimpse into an epigenetically controlled mechanism that links maternal high-fat diet with transgenerational susceptibility to alcohol abuse and anxiety.

**Electronic supplementary material:**

The online version of this article (10.1007/s00018-020-03705-6) contains supplementary material, which is available to authorized users.

## Background

In addition to their acute involvement in obesity [1, 2] there is evidence that diets high in fat are associated with an increase in anxiety and substance abuse in subsequent human generations [3, 4]. Consistent with these observations in humans, high-fat diet and maternal obesity is also associated with an increase in anxiety-like behaviour in mouse models. For example, decreased entries into the open arms of the elevated plus maze was observed in offspring of C57BL/6 mothers fed high-fat diet [5–7] and in Oldfield mice [8]. Later studies demonstrated that, in addition to increased anxiety-like behaviours, increased maternal HFD also altered the expression of a number of epigenetic regulatory molecules such as DNA methyltransferase 3a and b [9]. These studies raise the possibility that the effects of high-fat diet on the anxiety phenotype of subsequent offspring may have an epigenetic contribution. In addition, studies in rats suggest that maternal HFD can also influence intake of alcohol and nicotine in offspring [10]. Therefore, in addition to gaining an understanding of the molecular mechanisms regulating fat intake, anxiety and substance abuse there is also a priority to uncover the genomic and epigenetic mechanisms that contribute in linking high-fat diet to anxiety and substance abuse disorders.

Nutrient intake (fats and ethanol) and mood are controlled by the expression of neuropeptides in regions of the brain that include the hypothalamus and amygdala respectively [11]. The expression of one of these neuropeptides; galanin, a 30 amino acid peptide encoded by the *GAL* gene, contributes to the regulation of fat intake in animals [2, 12–14], plays a role in modulating mood [15–18] and regulates ethanol intake [19–22]. Considering its important role in these processes, much remains to be determined regarding the mechanisms that regulate the tissue-specific expression of the GAL gene. In order to address this knowledge gap we previously used comparative genomics to identify a highly conserved enhancer sequence (GAL5.1) that lay 42 kilobases (kb) 5′ of the *GAL* gene transcriptional start site in humans. We then demonstrated its activity in galanin expressing cells of the hypothalamus including the periventricular nucleus (PVN), dorsomedial hypothalamus (DMH) and arcuate nucleus (ARC) [23, 24]. The analysis of two polymorphisms in the human GAL5.1 enhancer using the UK Biobank cohort suggested a mechanistic link between allelic variants of these polymorphisms and alcohol abuse when stratified against sex (male) and anxiety [24]. Subsequent use of CRISPR genome editing, to disrupt the mouse GAL5.1 enhancer (mGAL5.1KO), demonstrated a role for mGAL5.1 in the tissue specific expression of the *Gal* gene in hypothalamus and amygdala as well as the modulation of alcohol intake and male anxiety-like behaviour [24].

Because increased susceptibility to anxiety and alcohol abuse occured in offspring of mothers fed a high-fat diet during pregnancy, we used the well characterised maternal high-fat diet [5–7] and maternal separation stress [25] paradigms in mice to explore the hypothesis that the anxiety and alcohol intake modulating GAL5.1 enhancer could be epigenetically influenced by environmental stimuli that include early life stress and high-fat diet. We also explored the effects of DNA methylation on GAL5.1 activity and its interactions with, and response to, known stimulators of GAL5.1 activity such as PKC agonism and EGR1 transcription factor expression. Finally, we asked whether deleting the GAL5.1 enhancer from the mouse genome using CRISPR genome editing has any effect on appetite, metabolism or selection of high-fat diet. We discuss our findings in the wider context of a role for GAL5.1 genetics and epigenetics in modulating fat intake as well as the possible role of altered GAL5.1 activity in increasing susceptibility to anxiety and alcohol abuse in future generations.

## Methods

### Animal studies

All animal studies were performed in full accordance with UK Home Office guidelines. Animals were maintained under specific pathogen-free (SPF) facilities. The health status of these animals conformed to The Federation of European Laboratory Animal Science Associations (FELASA) guidelines whereby pathogen screening was carried out on a quarterly (3 monthly) basis using sentinel animals. Male and female homozygous wildtype and mGAL5.1KO age matched littermates were single housed under standard laboratory conditions (12 h light/12 h dark cycle), in plastic cages with food and water available ad libitum, depending on the experiment.

### Epigenetic effects of early life stress by maternal deprivation

Wildtype C57BL/6 females were housed with wildtype C57BL/6 males and allowed ad-libitum access to standard CHOW diet. Once females became pregnant males were removed and females were allowed to litter down. Immediately after birth mothers were removed from the cages for two hours a day for the first 12 days as previously described [25]. Once weaned, litters were humanely sacrificed by euthatol injection and brain tissues (hypothalamus, hippocampus and amygdala) recovered and rapidly frozen on dry ice.

### High-fat diet studies

Wildtype C57BL/6 wildtype animals were housed with wildtype C57BL/6 males and allowed ad libitum access to a choice of low-fat diet (LFD; 22.03 kcal% protein, 68.9 kcal% carbohydrate and 9.08 kcal% fat) or high-fat diet (HFD; 20 kcal% protein, 20 kcal% carbohydrate and 60 kcal % fat; Research Diets Inc.) in different hoppers. Both hoppers were weighed regularly to ensure intake of HFD and LFD. Once females became pregnant males were removed and females allowed to litter down. After weaning (3-4 weeks), animals were humanely sacrificed by euthasol injection and brain tissues (hypothalamus, hippocampus and amygdala) recovered and rapidly frozen on dry ice.

### DNA extraction bisulphite conversion and pyrosequencing

**Fig. 1:**
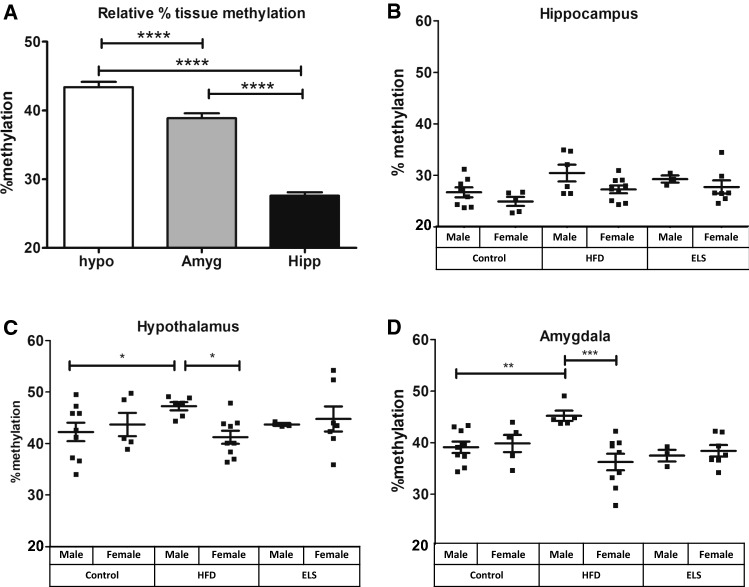
Levels of DNA methylation (5mC/5hmC) within GAL5.1 differs significantly between specific tissues and is affected by maternal access to high-fat diet, but not early life stress. **a** Bar plot demonstrating differential levels of 5mC/5hmC that occur within the GAL5.1 enhancer in DNA derived from hypothalamus (hypo), amygdala (amgd) and hippocampus (hipp) (*n* > 10). B–D Scatterplots showing the effects of high-fat diet (HFD) and early life stress (ELS) on 5mC levels of GAL5.1 derived from hippocampus (**b**), hypothalamus (**c**) and amygdala (**d**). n.s.; not significant, *; *p* < 0.05, **; *p* < 0.01, ***; *p* < 0.005

Genomic DNA was extracted and purified with the AllPrep DNA/RNA Mini Kit (Qiagen) and optimized for 10–15 mg brain tissue as per manufacturer’s instructions. The concentration and purity of genomic DNA was determined using a Nanodrop One^C^ (Thermo) and 500 ng of genomic DNA was bisulphite-converted using the EpiMark Bisulfite Conversion Kit (New England BioLabs) as per manufacturer’s instructions. The mouse GAL5.1 enhancer region analysed (chr19:3,441,054–3,441,445, GRCm38/mm10) contained 8 CpG sites (see Fig. 1). Primers were used to amplify two regions of the mouse Gal5.1 enhancer covering 8 CpGs: CpGs 4–8 (F–TTTAGTAGAGGAAATAAAATAGTAGAAAAA-Biotinylated; R: CCCCAAAAAACCACAAAACCTA) CpGs 9–11 (F – GGATGGAGGAATTTTTTTGTGTT; R: CCCCAAAAAACCACAAAACCTA -Biotinylated) using MyTaq HS mix PCR reagents (Bioline). Amplicons were processed on the Qiagen Q24 Workstation and sequenced in duplicate on the Qiagen Q24 pyrosequencer using the sequencing primers CpGs 4–8 (AACAATTTAAACAAAAAATAACATT) CpGs 9–11 (GAGTTTGATTGATAATAGTAGTAT) and DNA methylation levels across each CpG measured. These were expressed as percentage methylation.

### Reporter assays

The human GAL5.1 enhancer was cloned into the pCpGfree-promoter-lucia vector (InvivoGen; contains the EF1α promoter) and subjected to CpG methylation using SssI (New England Biolabs) as described in manufacturer’s instructions. Methylated and unmethylated plasmids were transfected into SHSY-5Y cells vector using Jetprime transfection reagent (Polyplus-transfection) and incubated for 24 h. Cells were lysed using passive lysis buffer and the activity of the enhancer assessed using a Lucia QUANTI-Luc Gold Assay #3 luciferase kit (InvivoGen). Lucia activity levels were normalised against quantitative PCR (qPCR) of levels of Lucia gene DNA present in cell extracts recovered using Qiagen PCR cleanup kits (Qiagen). Lucia luciferase activity was normalised against the crossing point (Cp) values generated from quantitative polymerase chain reaction (qPCR) of the lucia reporter vector. Absolute quantification of the transfected lucia reporter vector was determined in order to verify transfection efficiency between all samples. After media from cell wells was extracted, the cells were lysed using 100 µl passive lysis buffer (Promega) per well. Total DNA was isolated from the cell lysates using the QIAquick^®^ PCR Purification Kit (QIAGEN). The following primers used were designed to the lucia DNA sequence for amplification of the reporter gene (JE041; 5′- GAAATCAAGGTGCTGTTTGC-3′, JE042; 5′- TATCATCTGTCCCCAGCC-3′). Samples were loaded into an opaque 384-well plate in triplicate following the standard cycling parameters of a SYBR Green protocol with 60 °C annealing temperature. Amplification and quantitative analysis were carried out using the LightCycler^®^ 480 System (Roche).

### High-fat preference studies

Mice containing a disrupted GAL5.1 enhancer [24] were maintained as a colony on a heterozygous C57BL/6 background which was mated to produce homozygous wildtype and homozygous GAL5.1 KO age matched and sex matched individuals. These were identified using PCR of earclip DNA [24]. Once identified, animals were assigned random numbers to hide their genotype from the operators of subsequent tests. For high-fat diet preference studies, singly housed animals were provided with a choice of LFD or HFD in different hoppers. The position of each hopper was changed regularly to rule out the possibility of position effects. Animals were weighed daily over a period of 23 days and LFD and HFD were also weighed daily to determine intake of each diet.

### Metabolic analysis

Wildtype and GAL5.1KO animals were maintained for one week in sealed TSE metabolic cages where calorie intake (kcal/hour), O_2_ consumption (ml/hr), energy expenditure (kcal/hour) locomotor activity, CO_2_ production (ml/hour), energy balance (kcal/hr) respiratory exchange ratios and locomoter activity (beam breaks) were continuously monitored for 144 h with readings taken at 30 min intervals.

### Data analysis

From *in-vivo* pilot studies we calculated that a minimum of 6–8 animals per group would enable detection of a 25% difference between different parameters (e.g., high-fat diet, weight gain, metabolism) with 80% power using one-way ANOVA and/or general linear modelling. Statistical significance of data sets were analysed using either two way analysis of variance (ANOVA) analysis with Bonferroni post hoc tests or using one tailed or two tailed unpaired parametric Student *t* test as indicated using GraphPad PRISM version 5.02 (GraphPad Software, La Jolla, CA, USA).

## Results

We have previously shown that the GAL5.1 enhancer controls anxiety and alcohol intake [24]. This provided us with a unique opportunity to determine the effects of environmental factors such as dietary changes and early life stress on 5mC/5hmC levels within an enhancer region shown to control behaviours with a direct effect on human health. We first needed to establish normal levels of DNA methylation (5mC/5hmC) within GAL5.1 in different brain regions and to determine whether these levels differed between different brain regions.

### Levels of 5mC/5hmC within GAL5.1 varies significantly between brain regions

We first asked whether levels of 5mC/5hmC within GAL5.1 differed between different brain regions. To address this question, DNA from 3 different brain tissues (hypothalamus, amygdala and hippocampus) was recovered from mouse pups and bisulfite converted to detect levels 5mC/5hmC within GAL5.1 analysed using pyrosequencing (*n* > 10). We observed that 5mC/5hmC levels within GAL5.1 varied considerably between different brain tissues such that in hippocampus 5mC levels only reached 23–32% within GAL5.1. However, in amygdala and hypothalamus 5mC/5hmC levels were between 35 and 50% (Fig. 1a).

### 5mC/5hmC levels within GAL5.1 is not affected by early life stress

We next asked whether early life stress; an environmental factor known to affected enhancer 5mC/5hmC levels [25, 27] affected the methylation of the GAL5.1 enhancer in hypothalamus, hippocampus or amygdala. To address this question, we subjected newborn wildtype C57BL/6 mouse pups to maternal separation stress for 2 h a day for the first 12 days of their lives as previously described [25]. After weaning of these mice, DNA from 3 different brain tissues (hypothalamus, amygdala and hippocampus) was recovered, bisulphite converted and levels of 5mC/5hmC within GAL5.1 analysed using pyrosequencing. We did not observe any significant changes in 5mC/5hmC levels in any of the tissues recovered from animals subjected to early life stress (Fig. 1b-d).

### GAL5.1 displays significantly altered 5mC/5hmC levels in animals whose mothers had access to HFD

In order to address the possible role of high-fat diet in altering 5mC/5hmC levels within GAL5.1 we provided pregnant mice with a choice of low-fat or high-fat diet during pregnancy and during the rearing of their pups. Following weaning, pups were humanely sacrificed and genomic DNA was recovered from tissues dissected from hypothalamus, hippocampus and amygdala. This DNA was subjected to bisulphite conversion and pyrosequenced to determine changes in 5mC/5hmC levels within the GAL5.1 enhancer in the presence of access to HFD. We were unable to detect any changes in GAL5.1 5mC levels in DNA derived from hippocampal tissues of animals whose mothers had access to high-fat diet (Fig. 1b). However, significant changes in CpG methylation were observed in DNA derived from the hypothalamic regions of animals whose mothers had been exposed to high-fat diet such that there was a significant increase in methylation in males and a clear divergence in methylation between males and females (Fig. 1c). This increase in male GAL5.1 5mC levels and sexual divergence was more pronounced in DNA derived from amygdala (Fig. 1d).

### Increasing 5mC levels within the GAL5.1 enhancer suppresses its activity and its response to EGR1 binding and PKC activation

**Fig. 2:**
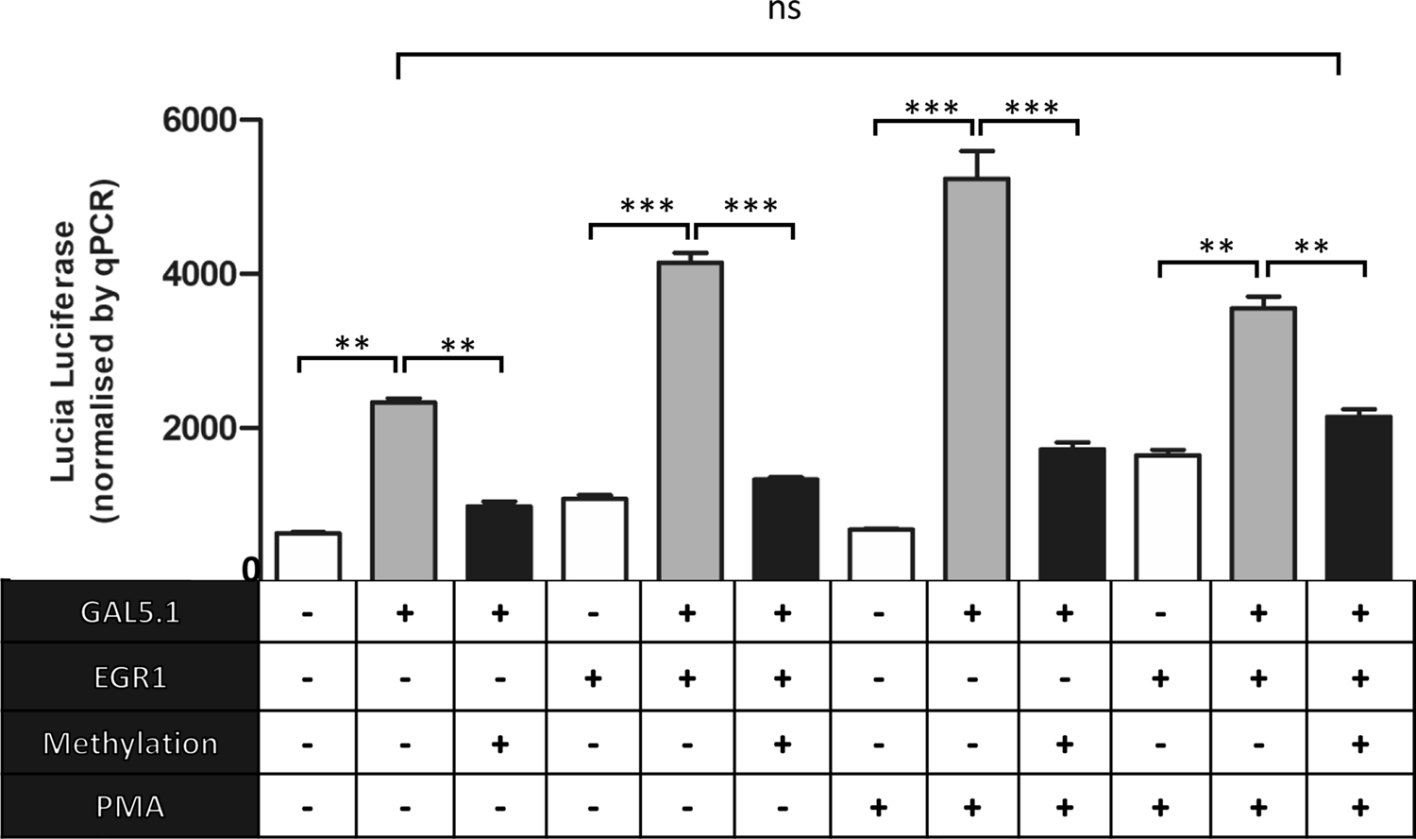
DNA methylation with SssI modulates the effects of PKC agonism and EGR1 transcription factor expression on the activity of the GAL5.1 enhancer. A bar graph demonstrating relative Lucia Luciferase activity of the pCpG-free vector (white bars), the pCpGfree-GAL5.1 (Grey bars) and pCpGfree-GAL5.1 methylated using SssI (Black bars) and co-transfected with the pcDNA-EGR1 expression vector (EGR1; bars 3–5 and 10–12) or treated with the PKC agonist PMA (PMA; bars 7–12). Each Lucia luciferase values was normalised against the relative quantities of Lucia luciferase DNA detected in each cell extract using quantitative PCR. *n* = 4, n.s.; not significant, **; *p* < 0.01, ***; *p* < 0.005

In addition to examining the effects of environmental factors on levels of 5mC/5hmC within GAL5.1 we also explored the effects of DNA methylation on the activity of GAL5.1 as well as its response to known stimuli such as PKC stimulation and expression of EGR1 [23, 24]. To address this question, we first cloned the human GAL5.1 (GG) enhancer into the pCpGfree-promoter-Lucia luciferase vector that lacks CpG dinucleotides and contains the EF-1α promoter. We then subjected this vector to CpG methylation using the bacterial SssI enzyme. Levels of plasmid methylation were monitored using digestion by the HpaII enzyme which is sensitive to methylated CpG sites (Figure S1). Methylated or unmethylated plasmid were then transfected into SHSY-5Y neuroblastoma cells in the presence of an expression vector expressing the EGR1 transcription factor (Fig. 2). These cells were then cultured for 24 h in the absence or presence of the PKC agonist PMA. Due to evidence of trans-interactions between our renilla control and the pCpG-lucia vector (probably through the EF-1α promoter), we devised an alternative transfection control based on quantitative PCR of the Lucia reporter gene. We observed that the GAL5.1 enhancer increased expression of Lucia luciferase compared with the empty vector but that this increase was negated by CpG methylation (Fig. 2). The previously reported stimulatory effects of either EGR1 co-expression or activation of the PKC pathway [23, 24] were similarly reduced by GAL5.1 methylation (Fig. 2). Interestingly, we observed that the activity of the non-methylated GAL5.1 enhancer was not significantly different from that of the methylated enhancer co-stimulated by both EGR1 expression and PKC activation (Fig. 2) suggesting that, together, EGR1 expression and PKC stimulation can overcome much of the effects of 5mC on GAL5.1 activity.

### mGAL5.1KO mice do not demonstrate significant changes in weight gain or intake of CHOW diet

**Fig. 3:**
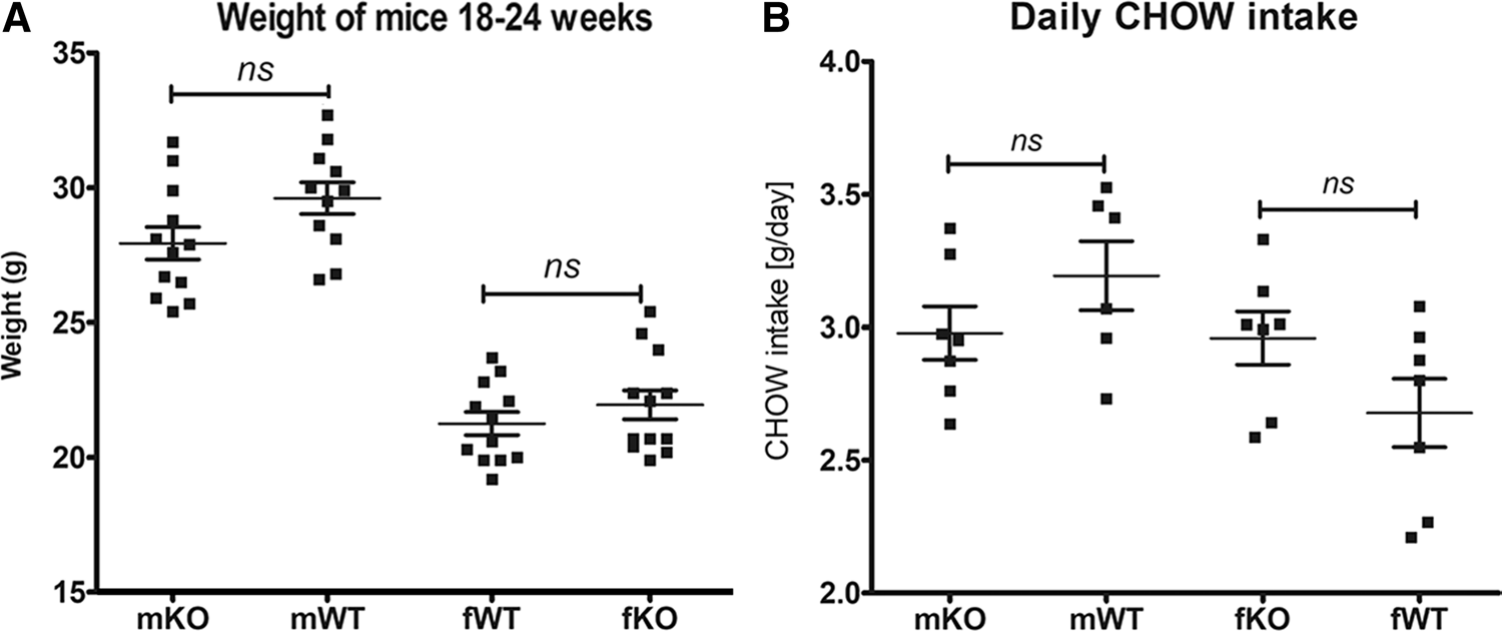
mGAL5.1KO animals do not differ significantly from wildtype animals in food intake or weight gain. **a** Scatterplots showing a comparison of weights of male (m) and female (f) mGAL5.1KO (KO) and wildtype (WT) animals at 24 weeks of age. **b** A comparison of daily CHOW intake of male and female wildtype and knock out animals. *ns* not significant

We have shown that maternal high-fat diet induces changes in levels of 5mC/5hmC within the GAL5.1 enhancer that we know controls anxiety like behaviour and ethanol intake in mice. We also know that deleting GAL5.1 greatly reduces the expression of the GAL gene [23, 24] in hypothalamus whose expression in this brain region controls fat intake [2, 12–14]. However, in order to explore the possibility that disrupting GAL5.1 had an effect on general appetite we first monitored food intake and weight gain of male and female homozygous wildtype (WT) and mGAL5.1KO animals provided with a standard CHOW diet. We found that, after 18–24 weeks, neither male nor female animals demonstrated significant differences in weight gain when compared with wildtype animals (Fig. 3a). We also found that GAL5.1KO animals ate similar levels of CHOW diet to WT animals (Fig. 3b) demonstrating that GAL5.1 did not control general appetite.

### mGAL5.1KO mice do not demonstrate significant metabolic differences to WT animals

**Fig. 4:**
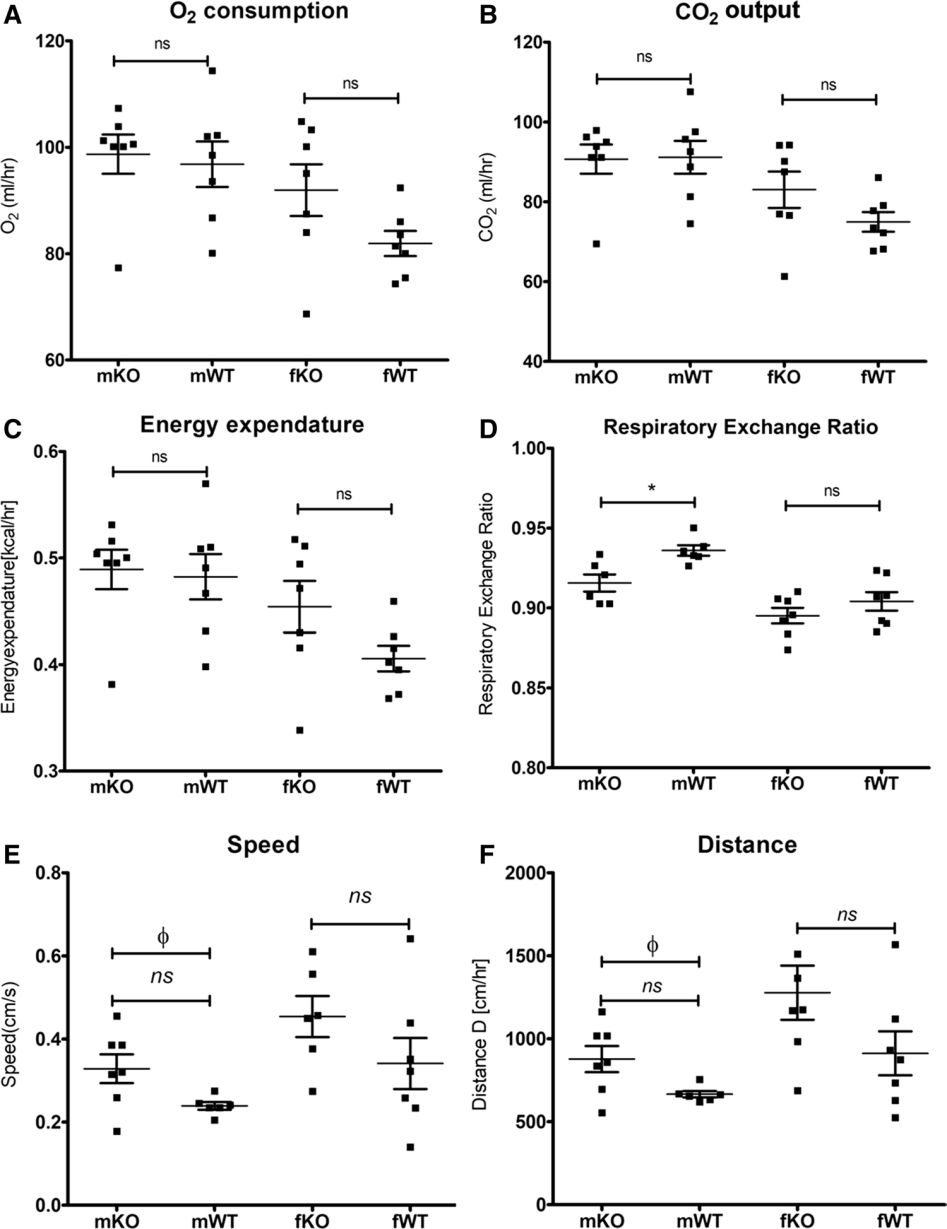
mGAL5.1KO mice do not differ significantly in their metabolism. **a**–**d**; Scatterplots showing a comparison of average O_2_ consumption (**a**), CO_2_ output (**b**), energy expenditure (**c**) and respiratory exchange ratio (**d**) in male (m) and female (**f**) mGAL5.1KO (KO) and wildtype (WT) animals recorded using sealed TSE cages. E, average speed (cm/second) and (**f**) distance travelled per hour (cm/hour). *; *p* < 0.05, *n.s.* not significant (ANOVA), *Φ* = significant (2-tailed *t* test)

In order to explore the possibility that disruption of GAL5.1 significantly altered metabolism we subjected mGAL5.1KO and wildtype male and female littermates to 116 h of metabolic analysis using sealed TSE cages that monitored variables such as O2 consumption, CO2 production (ml/hour), energy expenditure ration (kcal/hour) and energy balance (Kcal/hour). Over the 116 h of the analysis we detected little or no significant difference in O_2_ consumption (Fig. 4a), CO_2_ production (Fig. 4b) or energy expenditure (Fig. 4c) in male and female mice. however, we detected a significant decrease in the respiratory exchange ratio (Fig. 4d) in male animals that suggested higher activity levels. We observed an increase in distance travelled by male mGAL5.1KO animals (Fig. 4e) in addition to an increase in overall speed (Fig. 4f) consistent with higher levels of exploratory behaviour associated with the reduced anxiety phenotype previously reported [24].

### mGAL5.1KO mice exhibit decreased preference for high-fat diet

**Fig. 5:**
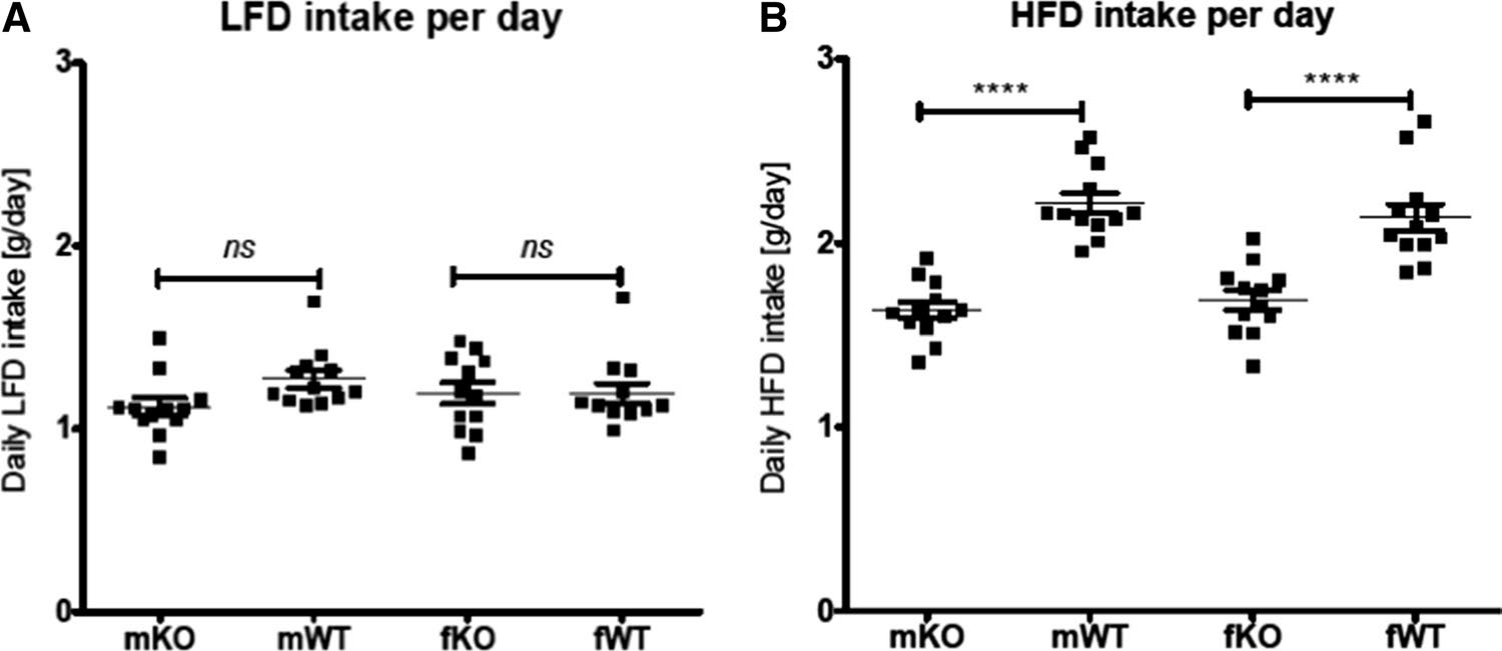
GAL5.1KO mice consume significantly less high-fat diet. **a** and **b**; scatterplots comparing daily intake in grams (g) of (**a**) high-fat diet and (**b**) low-fat diet comparing male (m) and female (f) GAL5.1KO (KO) and wildtype (WT) mice. *ns* = not significant, ****; *p* < 0.001

We demonstrate above that deleting the GAL5.1 enhancer had no significant effect on the intake of standard CHOW diet or the metabolism of animals fed this diet. However, previous studies demonstrated that deletion of exons 2–6 of the Gal gene in mice using ES-cell targeting [28] caused a significant reduction in the intake of high-fat diet by these animals compared with wildtype littermates but had no significant effects on protein or carbohydrate intake [14]. Because we have previously shown that disruption of the GAL5.1 enhancer resulted in a significant decrease of Gal mRNA expression in hypothalamus [24] we tested the hypothesis that CRISPR disruption of mGAL5.1 would affect preference for HFD in these mice. We provided singly housed, age- and sex-matched, littermate wildtype and mGAL5.1KO animals with a choice of low-fat diet (LFD; 6% of calories from fat) or high-fat diet (HFD; 60% of calories from fat) and monitored intake of LFD and HFD over 23 days. Both male and female mGAL5.1KO mice consumed significantly less HFD overall compared with wildtype littermates (Fig. 5a). Analysis of the total intake of LFD demonstrated no significant differences between the intake of wildtype and mGAL5.1KO animals (Fig. 5b). These experiments support the hypothesis that GAL5.1 is involved in driving a preference for high-fat food but not overall food intake.

## Discussion

Studies have suggested that increased maternal high-fat intake and obesity in both humans [3, 4] and animal models [5, 10] increase susceptibility to substance abuse and increased anxiety in resulting offspring. In the current study we explored the hypothesis that a contributing mechanism linking maternal high-fat diet and anxiety/substance abuse in offspring could involve the epigenetic modification of tissue specific enhancer regions through DNA methylation [29–31]. Our decision to explore a role for 5mC/5hmC in this process was based on the previous studies which showed that environmental challenges, such as high-fat diet and early life stress, change levels and distributions of 5mC/5hmC in the genomes of experimental animals [25, 32] and alter enhancer activity through interaction with DNA-binding proteins [33].

Because of its demonstrated role in controlling anxiety-like behaviour and ethanol intake, we focussed our attention on the CpG methylation of an enhancer sequence; called GAL5.1, which we have previously shown is responsible for supporting expression of the *Gal* gene in the hypothalamus and amygdala [23, 24]. We first asked whether environmental factors associated with poverty and deprivation in Western countries; including early life stress by maternal deprivation or consumption of high-fat diet, altered levels of DNA methylation within 8 CpG dinucleotides contained within the GAL5.1 enhancer sequence. We first noted significant differences in 5mC/5hmC levels in GAL5.1 derived from different tissues such that GAL5.1 from hippocampal tissues had the lowest 5mC/5hmC levels and hypothalamus and amygdala demonstrated the highest levels. These are interesting observations as the vast majority of large human cohort epigenome studies have based their analysis of levels and distribution of 5mC/5hmC on DNA derived from peripheral blood. However, given the observed differences in 5mC/5hmC levels observed between different brain tissues in the current study we suggest that caution should be exercised when interpreting studies based on the extrapolation of 5mC/5hmC levels in DNA derived peripheral blood to specific regions of the brain.

Although the repressive effects of 5mC on promoter activity through mechanisms involving the recruitment of methyl-CpG-binding proteins (MBD) and histone deacetylase (HDAC) containing complexes are well established and accepted [34, 35] the same cannot be said for remote tissue specific enhancer regions. For example, using DNAse hypersensitivity; a widely accepted marker of active enhancers, the ENCODE consortium only observed a decrease in chromatin accessibility in 20% of accessible sites as a result of CpG methylation suggesting that the remaining 80% of DNAseI hypersensitivity sites (markers of active enhancers) were still active despite increased 5mC levels [36]. Furthermore, an analysis of the effects of 5mC on the ability of 542 transcription factors (TFs) to bind DNA; an essential component of enhancer activation; found that only 23% of TFs experienced reduced binding as a result whereas the binding of 34% of TFs was actually increased by 5mC and the remaining TFs were unaffected [37]. The role of DNA methylation in influencing enhancer activity is further complicated by the influence of intermediate oxidation products of 5mC; that include 5-hydroxymethyl-cytosine (5hmC), the product of the ten-eleven translocation (TET) dioxygenase enzymes [38]. 5hmC sites are attractive to transcription factors that activate transcription [39]. 5hmC has been associated with active enhancers in stem cells [40, 41] and deletion of TET2 from mouse stem cells reduces 5hmC levels and activity of enhancers [41]. It is interesting, in this context, that the bisulphite sequencing protocol used in the current study is unable to differentiate 5mC from 5hmC so that it is as likely that the increased levels of 5mC observed within GAL5.1 as a consequence of maternal HFD intake actually reflects an increase in 5hmC levels. The possible role of 5hmC in upregulating enhancer activity, and our inability to differentiate 5mC and 5hmC using bisulphite conversion, may also explain the observations of increased 5mC/5hmC levels in hypothalamus and amygdala compared with the hippocampus that parallel the levels of Gal mRNA expression, which are significantly higher in hypothalamus and amygdala compared with hippocampus. Alternative methods of differentiating between genomic 5mC and 5hmC are now available which could help clarify the situation [42]. One fascinating possible contributory mechanism which might influence the changes in 5mC/5hmC levels observed in GAL5.1 due to HFD intake are the effects of HFD intake on gut microbiota [43]. Subsequent experiments will explore this possibility.

We designed a unique experiment to determine the effects of 5mC on the activity of the GAL5.1 enhancer and its response to EGR1 transcription factor binding and PKC agonism that we have shown stimulate activity of the GAL5.1 enhancer. To achieve this we cloned the GAL5.1 enhancer into a luciferase reporter vector (pCpGfree-lucia) that contained no CpG dinucleotides. This allowed us to selectively methylate CpG dinucleotides within the GAL5.1 enhancer, using the SssI enzyme, without affecting the vector backbone. These studies showed that CpG methylation of the GAL5.1 enhancer has a significant repressive effect on its activity in SHSY-5Y cells and confirmed previous studies demonstrating the repressive effects of 5mC on regulatory activity. However, our studies could not explore the effects of 5hmC on enhancer activity, which has been associated with increased enhancer activity [38–41]. Thanks to the recent availability of recombinant TET-proteins, that are able to convert 5mC to 5hmC, it may be possible to compare the effects of 5mC and 5hmC on GAL5.1 action in the near future [44].

Previous studies have shown that the binding affinity of the EGR1 transcription factor to DNA is not affected by 5mC [45]. However, we observed that 5mC suppressed GAL5.1 activity even in the presence of EGR1 expression that we have previously shown upregulated activity of the enhancer. This suggests that the binding of another transcription factor; who’s binding to DNA is affected by CpG 5mC, is critical to the normal functioning of GAL5.1. Identifying this transcription factor will be a major goal of subsequent analyses. 5mC CpGs within GAL5.1 also have a significant impact on the response of GAL5.1 to activation of the PKC pathway. Thus, the ability of 5mC to repress PKC activation of GAL5.1, even in the presence of EGR1, represents further evidence for the need to identify a second 5mC sensitive transcription factor in the normal response of GAL5.1. Interestingly, levels of activity of the fully methylated GAL5.1 enhancer in the presence of both EGR1 transcription factor expression and PMA do not differ significantly from that of the unmethylated GAL5.1 enhancer in the absence of these stimuli suggesting that the effects of 5mC on GAL5.1 might be overcome by increased TF binding and stimulation of signal transduction pathways in vivo*.* In the context of the role of the EGR1 transcription factor in modulating levels of 5mC/5hmC at the GAL5.1 locus it is also interesting that recent studies have identified EGR1 as a major recruiter of TET proteins to specific loci [46] a consideration which we will also consider in the design of future studies.

In the wider context of the relationship of maternal high-fat diet to increased susceptibility to substance abuse and anxiety, the current study raises the possibility of the mechanistic contribution of the GAL5.1 enhancer in this process. We have previously shown that GAL5.1 governs ethanol intake and anxiety-related behaviour in mice that is mirrored by a significant association between alcohol abuse and increased anxiety in humans [24]. In the current study we show that high-fat diet causes a significant change in 5mC/5hmC levels that are known to affect enhancer activity and that, in turn, GAL5.1 governs the decision to eat high-fat diet but also affects anxiety and alcohol intake. Taken together, we propose that maternal high-fat diet induced changes in GAL5.1 5mC/5hmC levels may alter GAL5.1 activity in subsequent generations in such a way as to affect the ability of GAL5.1 to affect anxiety and the decision to drink excess alcohol in subsequent generations.

## Conclusions

Whilst much remains to be done to differentiate the effects of environmental influence on the distribution of 5mC and 5hmC in GAL5.1, and how 5mC and 5hmC differentially affect GAL5.1 activity at a tissue specific level, the fact remains that we have identified a compelling epigenetic mechanism that may link the maternal intake of high-fat diet to the modulation of anxiety and alcohol intake in subsequent generations. These unique studies also provide an important stepping stone to discover the influence of environmentally modulated regulatory mechanisms in the development of neuropsychiatric disorders.

### Electronic supplementary material

Below is the link to the electronic supplementary material.

Supplementary file1 (PDF 170 KB)
