# Supplementary material for: The anxiety and ethanol intake controlling GAL5.1 enhancer is epigenetically modulated by, and controls preference for, high-fat diet
Source: Cell Mol Life Sci. 2020 Dec 12;78(6):3045–55. doi: 10.1007/s00018-020-03705-6 (PMC8004485; doi:10.1007/s00018-020-03705-6)
Supplement: Supplementary file 1 — Supplementary file1 (PDF 170 KB) [file 18_2020_3705_MOESM1_ESM.pdf]

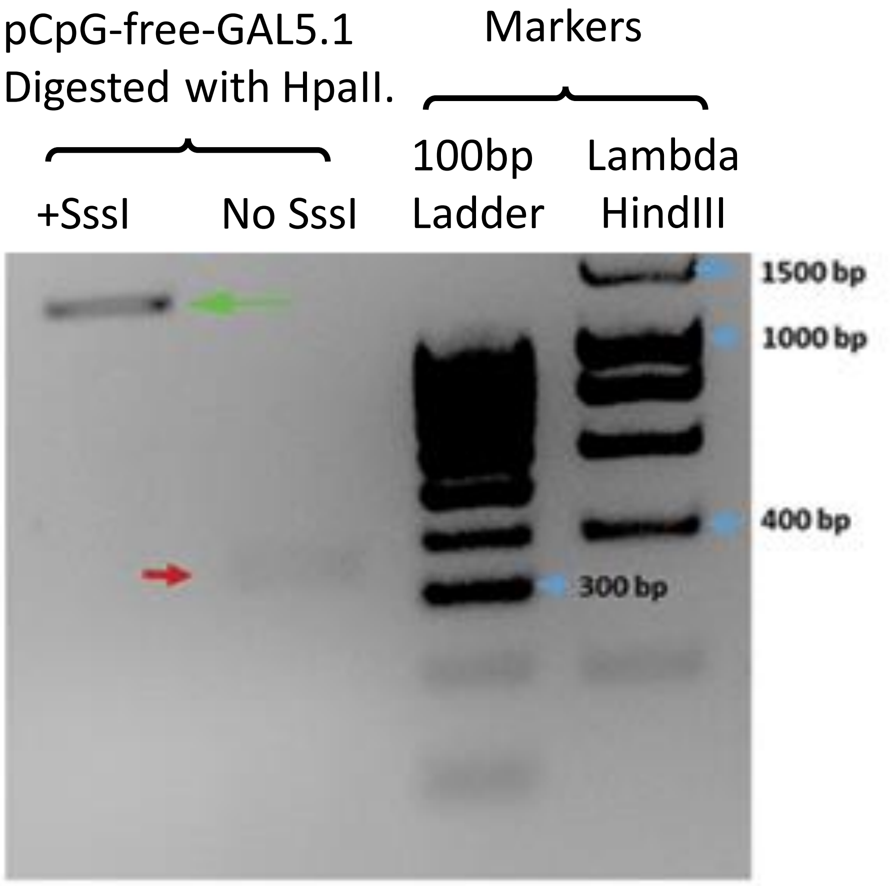

Supplementary data. A. DNA electrophoresis agarose gel demonstrating the effects of HpaII digestion on methylated (green arrow) and unmethylated pCpG-Free-GAL5.1 DNA (red arrow) demonstrating degrees of methylation. Blue arrows highlight relative sizes of DNA markers.
